# Partnering with *Life Science Alliance*

**DOI:** 10.15252/msb.20188327

**Published:** 2018-04-04

**Authors:** Thomas Lemberger

**Affiliations:** ^1^ EMBO Heidelberg Germany

## Abstract

*Molecular Systems Biology* is partnering with the new open‐access journal Life Science Alliance, an initiative by three major not‐for‐profit science organizations. An informed transfer process was established with our partner to provide authors with clear editorial commitments and reduce the overall burden on reviewers.

Portability and modularity are good engineering principles that are applied to build systems of increasing complexity with compatible components that do not need to be reinvented each time. These principles are, however, not always applied in scientific publishing where competing publishers tend to operate as independent ‘silos’ and have disappointingly little incentives to develop common standards for portable information exchange. This is particularly detrimental to the efficiency of the peer‐review process, where cohorts of reviewers are asked to spend a significant amount of time assessing the same manuscript over and over, as it goes through repeated submissions at a succession of journals. For the global research community, this results in a considerable waste of resources and slows down the publication process in the most frustrating way.

The idea of making peer‐review portable across journals is not new. Ten years ago, the neuroscience community launched the Neuroscience Peer Review Consortium (http://nprc.incf.org/) to enable transfer of reviews among participating journals. In addition, many journals offer authors the possibility to transfer their manuscripts and reviews to journals published by the same publisher. While these initiatives are welcome, they may not have always been as transformative as expected, in particular when applied across publishers. For one, it is difficult to change the behaviors of scientists and editors who often prefer to start the process from scratch rather than embarking on the reinterpretation of reviews written for another journal. But it may also reveal that portable review is only part of the solution. From our own experience with manuscript transfers within the EMBO Press journals, including *Molecular Systems Biology*, two factors are decisive to authors when they are considering whether to forward their manuscripts to another journal: obtaining a clear editorial commitment from the potential recipient journal; and keeping control over the fate of their work.

We are therefore delighted to announce the partnership of *Molecular Systems Biology* with the new open‐access journal *Life Science Alliance* (http://life-science-alliance.org), jointly published by EMBO Press, Rockefeller University Press and Cold Spring Harbor Laboratory Press. Together with the journals participating in this alliance, *Molecular Systems Biology* has established an informed transfer process that goes beyond the standard transfer of manuscripts with portable reviews. For manuscripts that cannot be accepted in *Molecular Systems Biology*, we will consult with the editors of *Life Science Alliance* before sending our decision. We will explicitly offer a transfer to authors if we obtain a strong commitment from *Life Science Alliance*. For studies that were peer‐reviewed at *Molecular Systems Biology*, the commitment of *Life Science Alliance* will be based on the available reviews and will include a set of clearly scoped revisions without the need to involve additional referees. Importantly, our authors will remain in control of the process: They can opt out of the informed transfer system at submission stage, and accepting or declining the offer to transfer is entirely up to them to decide.

The aim of this partnership is to accelerate the publishing process, and to make it more efficient, without sacrificing quality. Therefore, following good modular practice, the new *Life Science Alliance* “module” inherits many of the attributes from the partnering publishers. Policies and best practices that are already familiar to *Molecular Systems Biology* authors and readers are thus implemented at *Life Science Alliance*, including high standards for the quality of the peer‐review process, integration with preprint submission, protection against “scooping”, no reformatting required at submission, and publishing source data alongside figures. *Life Science Alliance* of course also accepts direct submissions, which undergo transparent peer review.

The alliance comprises nine major journals, including *Molecular Systems Biology*,* The EMBO Journal*,* EMBO Reports*,* EMBO Molecular Medicine*,* Journal of Cell Biology*,* Journal of Experimental Medicine*,* Journal of General Physiology*,* Genes and Development*, and *Genome Research*. In times when there is a risk of monopoly by few publishers dominating the whole landscape, we feel proud to be part of an alliance, whose members have put aside the inherent competition among their individual journals to join forces in the interest of the research community.

